# Systematic review and meta-analysis of T1 glottic cancer outcomes comparing CO_2_ transoral laser microsurgery and radiotherapy

**DOI:** 10.1186/s40463-019-0367-2

**Published:** 2019-09-03

**Authors:** Michael F. Vaculik, Colin A. MacKay, S. Mark Taylor, Johnathan R. B. Trites, Robert D. Hart, Matthew H. Rigby

**Affiliations:** 1 Dalhousie Medical School, Dalhousie University, Halifax, Nova Scotia Canada; 2 Dalhousie Division of Otolaryngology – Head and Neck Surgery, Dalhousie University, Suite 3044, Dickson Bldg. 5820 University Avenue, Halifax, NS B3H 1V9 Canada

**Keywords:** Early glottic cancer, Transoral laser microsurgery, Radiotherapy, Survival

## Abstract

**Background:**

The objective of this study is to compare the oncologic outcomes of CO_2_ transoral laser microsurgery (TLM) and radiotherapy (RT) for treatment of T1 glottic carcinoma.

**Methods:**

A literature search was conducted in the following databases: Medline/PubMed, Web of Science, EMBASE, and the Cochrane Library. Search results were screened, and publications comparing oncologic outcomes of T1N0M0 glottic carcinoma treated with TLM or RT were included. Data was extracted independently by two authors, and publication quality was graded according to the Oxford Centre for Evidence-based Medicine. Meta-analysis was performed for overall survival, disease specific survival, laryngeal preservation, and local control.

**Results:**

Sixteen studies were included in the meta-analysis, the majority being retrospective cohort studies with two prospective cohort studies. Included studies were rated as either Level II or III evidence. Meta-analysis favoured treatment with TLM for T1 glottic carcinoma patients for the following outcomes: overall survival (odds ratio [OR], 1.52; 95% confidence interval [CI], 1.07–2.14; *P* = 0.02), disease specific survival (OR, 2.70; CI, 1.32–5.54; *P* = 0.007), and laryngeal preservation (OR, 6.31; CI, 3.77–10.56; *P* < 0.00001). There was no difference in local control between TLM and RT in T1 glottic cancer (OR, 1.19; CI, 0.79–1.81; *P* = 0.40).

**Discussion:**

Our study provides a current and thorough comparison of TLM and RT outcomes in T1 glottic carcinoma. Limitations of our study include lack of randomized control trials, and non-randomized allocation of patients to treatment groups. Our meta-analysis suggests that TLM is the superior modality in terms of overall survival, disease specific survival, and laryngeal preservation. Future prospective randomized controlled studies are required for confirming these findings and developing appropriate clinical practice guidelines.

**Level of evidence:**

2A; as per the Centre of Evidence Based Medicine.

## Introduction

Cancer of the larynx is the most common head and neck malignancy, with an estimated 1150 new cases in Canada in 2017 [1]. In recent years, incidence rates in both men and women have declined, which reflects a reduction in the use of proven risk factors including cigarettes and alcohol. Nevertheless, about 440 Canadians died from laryngeal cancer in 2017 [1]. About two-thirds of laryngeal cancers arise in the glottic area, the majority of which are diagnosed early, partly due to larynx anatomy, specifically its encasement in cartilage with sparse lymphatics, and partly due to early onset of symptoms including hoarseness. Early diagnosis provides an important opportunity for organ preservation and cure. As a result, the selection of optimal management is crucial in maximizing oncologic and functional outcomes.

In the past, laryngeal cancers were traditionally treated with radiotherapy (RT) or open partial laryngeal surgery. Transoral laser microsurgery (TLM) for glottic cancer was first described in 1972 by Strong and Jako [2], and later popularized in 1988 by Steiner [3]. Currently, TLM and RT are both acceptable treatment modalities for early glottic cancer, with open surgery now falling out of favour [4]. It is evident that these therapies may differ in side-effect profile and cost in the short term, however, there remains debate on which results in better functional and oncologic outcomes [5–7]. At present, there is a paucity of high quality prospective evidence in academic literature directly comparing TLM and RT in early glottic cancer. According to a recent Cochrane review, there is just one randomized-control trial (RCT) published in 1990 that compares TLM, open surgery, and radiotherapy in 234 patients with early glottic cancer [8]. This study, however, does not offer sufficient evidence to guide clinical decision making. As a result, surgeons must rely on the critical appraisal of non-randomized studies with variable patient populations, cancer staging, and outcomes. Adding to the complexity of therapy selection is patient preference, clinical characteristic, and availability of either modality across institutions. Predictably, current opinions of optimal therapy differ across disciplines and countries [9, 10].

To our knowledge, an international standard guiding therapeutic selection in early glottic carcinoma does not exist. In order to improve clinical decision making, we systematically reviewed the literature for studies directly comparing TLM and RT in T1 glottic carcinoma and performed a meta-analysis of critical oncologic outcomes including overall survival, disease-specific survival, laryngeal preservation, and local control. Although previous reviews have investigated this question, our systematic review provides an updated analysis. For instance, Mo et al. in 2017 [6] includes eleven studies published before January 2012. In comparison, our systematic review includes sixteen studies, five of which [11–15] published after January 2012. We also excluded single arm studies from our review, whereas Higgins et al. [16] assessed overall survival by exclusively comparing single arm TLM and RT studies. As a result, our analysis provides a current and rigorous review of this question.

## Methods

### Literature search strategy

Literature searches were conducted independently by authors MFV and CAM in the following electronic databases: Medline/PubMed, Web of Science, EMBASE, and the Cochrane Library. These databases were selected to provide a broad search of high quality medical research and allowed for a thorough scope of the available literature. Searches were designed to combine disease specific terms (neoplasm, cancer, malignan*, carcinoma*, tumour*, tumor*, SCC), site specific terms (larynx, laryn*, glott*, throat, ‘voice box’, ‘vocal cord’), and treatment specific terms for TLM (surgery, ‘larynx surgery’, ‘transoral laser microsurgery’, ‘trans oral laser microsurgery’, ‘endoscopic surgery’, microsurgery, ‘CO2 laser’, ‘carbon dioxide laser’), and RT (radiotherapy, radiotherap*, irradiat*, radiat*, ‘rt’, radiation). Searches in separate databases were similarly constructed, using the Boolean operator ‘AND’ to combine disease, site, and treatment specific term categories. All searches were conducted between March and May 2017.

### Inclusion and exclusion criteria

Search results for each database were imported in EndNote X8.2 software by Clarivate Analytics. According to currently accepted practises, duplicate results between databases were retrieved using the “Find Duplicates” function in EndNote X8.2, and manually reviewed by author MFV prior to removal [17]. Titles and abstracts were then screened separately by authors MFV and CAM for the following inclusion criteria (1): patient with untreated T1N0M0 glottic carcinoma (2); comparative cohorts that received a primary treatment with either carbon dioxide TLM, or RT (3); analysis of one or more oncologic outcomes including overall survival, disease specific survival, local control rate, locoregional control rate, and laryngeal preservation rate. Studies were excluded if any of the following criteria were met (1): glottic cancer other than stage T1 (2); studies reporting only on functional results or voice quality (3); studies using KTP laser for TLM treatment; and (4) single-arm studies reporting on one of the two therapy methods, or (5); studies with incomplete data. Selected studies had their bibliographies cross-referenced for any unidentified publications. Database searches also retrieved systematic and other literature reviews, whose bibliographies were independently screened by both MFV and CAM for additional studies meeting inclusion criteria. For differences in inclusion between screening authors, a discussion was held and consensus reached on inclusion or exclusion. Only abstracts in English were reviewed. Some non-English studies were retrieved in the search however they did not meet the stated the inclusion criteria. Finally, when data were felt to have been duplicated by authors in more than one manuscript, the most recent study was included for the current review.

### Data extraction

Full texts of eligible studies according to the above inclusion criteria were retrieved and carefully searched independently by authors MFV and CAM. For each text, study characteristic data was extracted and reported in Table 1, including year of publication, location of study, study design, study period for data collection, stages of glottic cancer included, staging system if reported, study comparison arms, follow-up time for which data is reported, mean age of participants and age range if reported, and the quality assessment score. Sample size and oncologic outcome data was extracted and reported in Table 2. Oncologic outcomes, including overall survival, disease free survival, local control, and laryngeal preservation are reported as events to facilitate meta-analysis. Percentages displayed in Table 2 were calculated as events over sample size, and are not the reported Kaplan Meier data. Not all eligible studies reported data on all of the oncologic outcome measures. Additionally, data for oncologic outcomes was not included if only Kaplan Meier percentages were reported. For studies that reported on oncologic outcomes of stages other than T1, data was included if authors specified outcomes by stage. If differences in data extraction results occurred between authors, a consensus was reached through discussion with MHR.

**Table 1:** Characteristics of the included studies

Study	Year	Country	Design	Study Period	Cancer Staging	Arms	Stages	Sample Size	Sex (M:F)	Mean Age (range)	Selection Bias	ELS Type	Quality Score
Low et al. [11]	2017	Canada	retro	2003–2013	NR	TLM, RT	T1a	105	86:19	68	NR	NR	Level III
Alkan et al. [12]	2017	Israel	retro	2006–2013	NR	TLM, RT	T1a T1b	23 31	46:8	68 (36–95)	AC, Age, Tumor	IV / V	Level III
Taylor et al. [13]	2013	Canada	pro	2002–2010	NR	TLM, RT	T1b	63	57:6	67	NR	NR	Level II
Remmelts et al. [14]	2013	Netherlands	retro	2000–2008	200;2 UICC	TLM, RT	Tis T1a T1b T2	26 103 42 77	88:12	65 (39–89)	NR	NR	Level III
Kerr et al. [15]	2012	Canada	retro	2002–2010	NR	TLM, RT	T1a T1b T2	146 41 46	205:29	67	NR	NR	Level III
Kujath et al. [20]	2011	Canada	retro	2000–2009	NR	TLM, RT	T1 T2	64 15	69:10	NR	Time Period	NR	Level III
Mahler et al. [25]	2010	Norway	pro	1986–2005	2002 UICC	TLM, RT	T1a	351	318:33	66 (31–90)	Time period	NR	Level II
Schrijvers et al. [26]	2009	Netherlands	retro	1990–2004	2002 AJCC	TLM, RT	T1a	100	88:12	66 (38–83)	Time period	I / II	Level III
Thurnher et al. [28]	2008	Austria	retro	1948–1997	2002 UICC	TLM, RT, PL	T1a	337	309:28	63 (28–90)	NR	NR	Level III
Sjögren et al. [23]	2008	Netherlands	retro	1996–2007	NR	TLM, RT	T1a	181	165:15	70 (33–95)	AC	I / II	Level III
Goor et al. [22]	2007	Netherlands	retro	1995–1999	NR	TLM, RT	T1a	89	85:4	65 (42–83)	Tumor (Depth)	II	Level III
Krengli et al. [18]	2004	Italy	retro	1990–2001	NR	TLM, RT	T1a	57	55:2	68 (55–81)	NR	III / IV	Level III
Stoeckli et al. [19]	2003	Switzerland	retro	1990–1998	1997 UICC	TLM, RT	T1 T2	101 39	132:8	63 (41–88)	Stage, Tumor	NR	Level III
Brandenburg [21]	2001	USA	retro	1989–1999	NR	TLM, RT	T1a T1b	70 4	65:9	64 (33–88)	AC	NR	Level III
Rosier et al. [24]	1998	Belgium	retro	1979–1995	1987 UICC	TLM, RT, PL	T1a T1b	81 25	93:13	64 (43–88)	Age, AC, Stage	NR	Level III
Epstein et al. [27]	1990	USA	retro	1975–1987	1988 AJCC	TLM, RT	T1a	77	71:6	63	NR	NR	Level III

*Abbreviations: AC* anterior commissure*, AJCC* American joint committee on cancer*, ELS* European laryngological society*, F/U* follow-up*, NR* not reported*, PL* partial laryngectomy*, pro* prospective*, RT* radiotherapy*, retro* retrospective*, TLM* transoral laser microsurgery*, UICC* Union for international cancer control

**Table 2:** Survival outcomes of the included studies

Study	Stages Included in Analysis	Mean Follow-Up Time	Sample Size		OS % (n)		DSS % (n)		LP % (n)		LC % (n)	
			TLM	RT	TLM	RT	TLM	RT	TLM	RT	TLM	RT
Low et al. [11]	T1a	5 yrs	53	52	89 (47)	87 (45)	100 (53)	98 (51)	100 (53)	92 (48)	81 (43)	92 (48)
Alkan et al. [12]	T1a, T1b	5 yrs	16	38	88 (14)	79 (30)	100 (16)	97 (37)	94 (15)	89 (34)	75 (12)	87 (33)
Taylor et al. [13]	T1b	2 yrs	21	42	90 (19)	90 (38)	95 (20)	98 (41)	100 (21)	90 (38)	95 (20)	88 (37)
Remmelts et al. [14]	T1a	5 yrs	50	54	90 (45)	81 (44)	100 (50)	98 (53)	100 (50)	96 (52)	90 (45)	94 (51)
Kerr et al. [15]	T1a, T1b	2 yrs	125	62	94 (118)	95 (59)	–	–	100 (125)	92 (57)	–	–
Kujath et al. [20]	T1	3 yrs	51	46	–	–	100 (51)	100 (46)	100 (51)	91 (42)	–	–
Mahler et al. [25]	T1a	3 yrs	188	163	88 (165)	81 (132)	98 (184)	97 (158)	99 (187)	93 (152)	93 (174)	89 (145)
Schrijvers et al. [26]	T1a	5 yrs	49	51	92 (45)	82 (42)	100 (49)	98 (50)	96 (47)	80 (41)	73 (36)	76 (39)
Thurnher et al. [28]	T1a	5 yrs	81	108	–	–	100 (81)	91 (98)	100 (81)	84 (91)	90 (73)	69 (75)
Sjögren et al. [23]	T1a	5 yrs	73	70	84 (61)	80 (56)	100 (73)	96 (67)	100 (73)	83 (58)	89 (65)	79 (55)
Goor et al. [22]	T1a	2 yrs	54	31	100 (54)	100 (31)	100 (54)	100 (31)	100 (54)	97 (30)	94 (51)	90 (28)
Krengli et al. [18]	T1a	5 yrs	122	80	–	–	–	–	96 (117)	91 (73)	96 (117)	91 (73)
Stoeckli et al. [19]	T1	5 yrs	56	45	91 (51)	89 (40)	96 (54)	96 (43)	96 (54)	82 (37)	88 (49)	82 (37)
Brandenburg [21]	T1a, T1b	5 yrs	30	44	–	–	100 (30)	95 (42)	97 (29)	86 (38)	83 (25)	80 (35)
Rosier et al. [24]	T1a, T1b	5 yrs	31	41	–	–	–	–	–	–	84 (26)	90 (37)
Epstein et al. [27]	T1a	5 yrs	17	43	–	–	–	–	88 (15)	84 (36)	71 (12)	86 (37)

*Abbreviations: RT* radiotherapy*, TLM* transoral laser microsurgery, *OS* overall survival, *DSS* Disease Specific Survival*, LC* local control*, LP* laryngeal preservation*, retro* retrospective*, pro* prospective

### Retrieval results

**Fig. 1:**
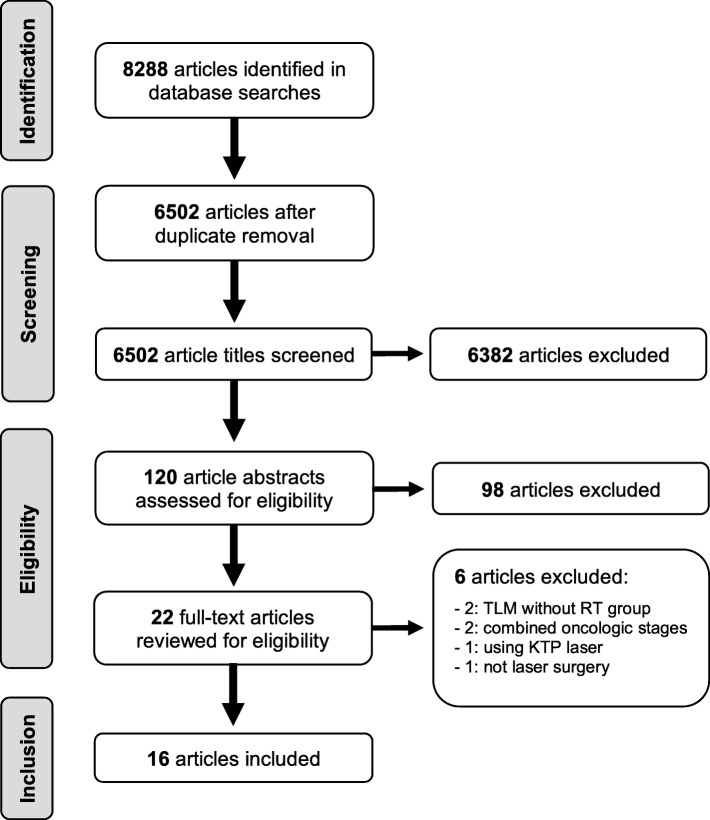
PRISMA flow diagram of literature search and selection process

The search strategy retrieved 6502 unique studies, whose title, keywords, and abstract if necessary were reviewed independently by MFV and CAM. A total of 120 abstracts were selected for analysis and were subsequently narrowed to 41 full-text articles (including 19 literature reviews). After comprehensive review, we found only 14 eligible studies from our search, with two additional studies retrieved from analyzing references of literature reviews. The final result was 16 eligible studies that met inclusion criteria [11–15, 18–28], and all reported baseline characteristics summarized in Table 1. The steps that were followed to identify the appropriate studies are illustrated in Fig. 1.

### Study characteristics

Of the sixteen eligible studies in our meta-analysis, ten were published within the last decade, and of these only six [11–15, 20] performed cancer treatment within this time period. Reporting of patient baseline characteristics varied greatly. The total number of patients in our meta-analysis pooled from all eligible studies was 1987, 1017 for the TLM arm and 970 for the RT arm, respectively. Five studies [11, 14, 20, 23, 26] had a balanced sample size in both treatment arms, whereas eleven studies [12, 13, 15, 18, 19, 21, 22, 24, 25, 27, 28] had an unbalance sample size, of which just one study [25] reported an analysis for statistically significant differences in baseline patient characteristics between treatment arms. Eight studies [11, 15, 18, 19, 22, 23, 25] had a larger sample size in the TLM arm compared to the RT arm. All studies, except for one [20], reported the mean age of participants, which was similar overall and ranged from 63 to 70 years.

In terms of glottic cancer staging and treatment, just seven studies reported use of a specific staging system, of which five studies [14, 19, 24, 25, 28] used the Union for International Cancer Control (UICC) staging system and two studies [26, 27] used the American Joint Committee on Cancer (AJCC) staging system. For TLM, just four studies reported laser parameters, including laser power used during surgery, with two studies [11, 25] reporting a laser power of 1–2 W, one with 2–9 W laser power [14], and another with a large range of 0.5–55 W of power [24]. Additionally, just five studies [12, 18, 22, 23, 26] reported the type of cordectomy performed according to the European Laryngological Society [29]. Briefly, type of cordectomies are classified as follows: type I is a subepithelial cordectomy; type II is a subligamental cordectomy; type III is a transmuscular cordectomy; type IV is a total cordectomy; and type V is an extended cordectomy. There was variability amongst the type of cordectomy performed for T1 glottic cancer, including two studies [23, 26] that performed type I or II, one study [22] that performed solely type II, one study [18] that performed type III or IV, and one study [12] that performed type IV or V. In terms of RT, most studies [11, 12, 14, 18, 19, 21–28] reported dose and treatment regimens, with the average dose ranging from 60 to 73 Gy. The source of radiation was most often a 6-MV linear accelerator. Some studies used a 4-MV or 18-MV linear accelerator [24, 27], x-rays [28], or a Co60 unit [24, 27, 28].

The majority of studies reported oncologic outcomes for T1a glottic cancer, with five studies [12, 15, 21, 24] reporting outcomes for both T1a and T1b, and one study [13] reporting outcomes solely for T1b. Four studies [15, 19, 20, 24] also reported separate outcomes for T2 glottic cancer in both treatment arms that was not included in our meta-analysis. Lastly, two studies [24, 28] had additional partial laryngectomy treatment arm that was also not included in our analysis. The majority of follow-up time points for reported oncologic outcomes was 5 years. Two studies [20, 25] reported oncologic outcomes at 3 years, and three [13, 15, 22] reported outcomes at 2 years.

### Methodological quality

A methodological quality assessment of eligible studies was performed using a grading tool published by Oxford Centre for Evidence-based Medicine [30] as per previously published methods in the field of otolaryngology [6, 31]. Additional factors were considered, including the transparency, clarity, and extent of outcome data reported, the inclusion of relevant patient baseline characteristics, clearly defined methods in staging and treatment, and presence of selection bias. There were no eligible RCTs or randomized studies for inclusion. Thus, the body of evidence in this systematic review is comprised entirely of non-randomized observational cohort studies, fourteen retrospective and two [13, 25] prospective in design. As a result, the majority of eligible studies were graded as Level III evidence, while 2 studies [11, 25] were graded as Level II. Eight of sixteen studies reported potential sources of selection bias, including age of patient [12, 24], anterior commissure (AC) involvement [12, 21, 23, 24], tumor characteristics [12, 19, 22], variable treatment time periods [20, 25, 26], and TNM stage [19, 24].

### Statistical analysis

Event numbers of oncologic outcomes from both TLM and RT treatment arms were pooled from eligible studies. Meta-analysis was performed using RevMan 5.3, an open-source statistical analysis software (The Cochrane Collaboration, Oxford, UK). Heterogeneity between studies was assessed via a chi-square analysis and the I [2] test, with significance set at *P* < 0.1. Included studies are considered to have low heterogeneity (or be homogeneous) if I [2] is less than 25%, moderate heterogeneity if I [2] is 25 to 50%, and high heterogeneity if I [2] is greater than 50%. If homogeneity existed between studies, meta-analysis was performed with a fixed effect model. If significant heterogeneity was confirmed, either by significant chi-square test (*P* < 0.1) or I [2] greater than 50%, meta-analysis was performed using a random effects model. Lastly, a pooled odds ratio (OR) was performed with 95% confidence interval (CI), and the overall effect was assessed via the *z* statistic with statistical significance set at *P* <0.05.

## Results

### Oncologic outcomes

Oncologic outcomes for included studies are reported as number of events with sample size for reference in Table 2. All 16 eligible studies were included in at least one oncologic outcome meta-analysis. The majority of studies reported laryngeal preservation and local control as primary outcomes.

#### Overall survival

**Fig. 2:**
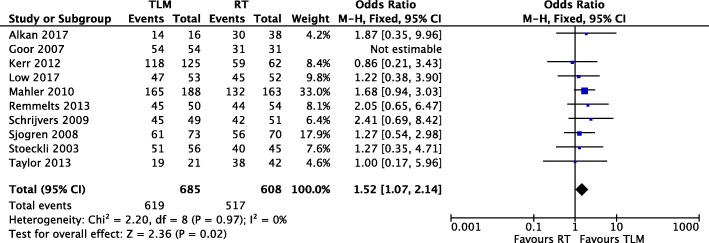
Forest plot comparison of TLM and RT in T1 glottic cancer with respect to overall survival

Of the sixteen studies included in our meta-analysis, ten [11–15, 19, 22, 23, 25, 26] reported overall survival event numbers for both TLM and RT treatment arms (Fig. 2). The total patient population was 685 in the TLM arm and 608 in the RT arm. One study [22] reported 100% overall survival in both the TLM and RT treatment arms and thus could not be included in the analysis. Meta-analysis revealed low heterogeneity among the eight retrospective and two prospective cohort studies (Chi^2^ = 2.20, *P* = 0.97, I^2^ = 0%), and the fixed effect model was applied. The pooled analysis significantly favoured TLM for overall survival of T1 glottic cancer patients, with an OR of 1.52 (95% CI of 1.07, 2.14) and a Z score of 2.36 (*P* = 0.02).

#### Disease-specific survival

**Fig. 3:**
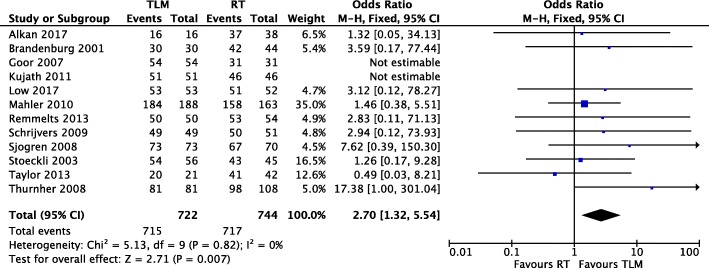
Forest plot comparison of TLM and RT in T1 glottic cancer with respect to disease-specific survival

Twelve of sixteen studies [11–14, 19–23, 25, 26, 28] reported event numbers for disease specific survival for both TLM and RT treatment arms (Fig. 3). There were 722 patients in the TLM arm and 744 in the RT arm. Two studies [20, 22] reported 100% disease-specific survival in both the TLM and RT treatment arms and were not included in the analysis. There was low heterogeneity among the ten retrospective and two prospective cohort studies (Chi^2^ = 5.13, *P* = 0.82, I^2^ = 0%), and the fixed effect model was applied. Meta-analysis significantly favoured TLM for disease-specific survival in T1 glottic cancer, with an OR of 2.70 (95% CI of 1.32, 5.54) and a Z score of 2.71 (*P* = 0.007).

#### Laryngeal preservation

**Fig. 4:**
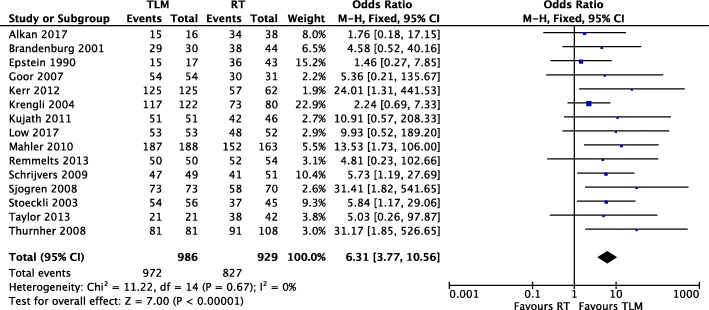
Forest plot comparison of TLM and RT in T1 glottic cancer with respect to laryngeal preservation

Fifteen of sixteen studies [11–15, 18–23, 25–28] reported event numbers for laryngeal preservation for both TLM and RT treatment arms (Fig. 4). There were 986 patients in the TLM arm and 929 in the RT arm. There was low heterogeneity among the thirteen retrospective and two prospective cohort studies (Chi^2^ = 11.22, *P* = 0.67, I^2^ = 0%), and the fixed effect model was applied. Meta-analysis significantly favoured laryngeal preservation with TLM, with an OR of 6.31 (95% CI of 3.77, 10.56) and a Z score of 7.00 (*P* < 0.00001).

#### Local control

**Fig. 5:**
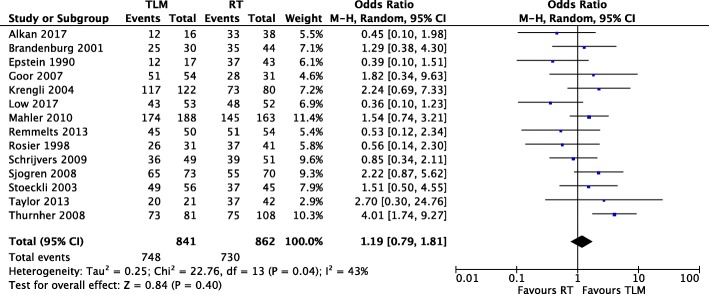
Forest plot comparison of TLM and RT in T1 glottic cancer with respect to local control

Fourteen of sixteen studies [11–15, 18–23, 25–28] reported local control event numbers for both TLM and RT treatment arms (Fig. 5). There were 841 patients in the TLM arm and 862 in the RT arm. There was significant heterogeneity among the twelve retrospective and two prospective cohort studies (Chi^2^ = 22.76, *P* = 0.04, I^2^ = 43%), and the random effects model was applied. Meta-analysis demonstrated no difference in local control with TLM or RT as primary therapy, with an OR of 1.19 (95% CI of 0.79, 1.81) and a Z score of 0.84 (*P* = 0.40).

## Discussion

Since TLM was introduced in 1972 [2], it has become a preferred therapeutic modality for early glottic cancer. Due to a paucity in high quality research, currently there are equivalently acceptable tools in our armamentarium, including RT. The lack of randomized prospective studies directly comparing TLM and RT has complicated clinical decision making, forcing surgeons to rely upon non-randomized studies. To contribute to improved clinical decision making, we conducted a current and thorough systematic review and meta-analysis of published literature in major electronic databases that assessed oncologic outcomes of T1 glottic cancer patients treated with TLM or RT.

### Oncologic outcomes

Meta-analysis revealed the absence of heterogeneity in all oncologic outcomes with the exception of local control. This was mitigated by the use of a random effects model rather than a fixed effects model in our meta-analysis. To our knowledge we are the first systematic review comparing TLM and RT in early glottic cancer to demonstrate a significant improvement in disease-specific survival with TLM (Fig. 3). In addition, our study shows that TLM is strongly favourable for organ preservation, with an OR of 6.31 for laryngeal preservation in TLM versus RT (Fig. 4). With TLM, patients are therefore approximately six times more likely to preserve their larynx than those treated with RT. This finding is in agreement with three previous systematic reviews with meta-analyses [5, 6, 32]. In terms of overall survival, debate exists as to whether TLM is advantageous as compared to radiotherapy. Two systematic reviews support this notion [6, 16], while two others do not find a significant difference [5, 32]. With the inclusion of recent studies, we demonstrate that treatment with TLM is associated with improved overall survival over RT in early glottic cancer (Fig. 2). Lastly, we did not demonstrate a significant difference between TLM and RT in local control outcomes of early glottic cancer (Fig. 5). This finding is consistent with multiple systematic reviews published within the last decade [5, 6, 16, 32–34]. Overall, our results confirm that primary therapy with TLM is equally efficacious as RT in local control after an initial resection, but offers an advantage in laryngeal preservation, overall survival, and disease-specific survival. This advantage could arise from the ability to precisely resect lesions and conserve surrounding anatomy with TLM. In this respect, initial TLM does not preclude further use of TLM in the management of local recurrence, and RT remains a viable option. All studies used first recurrence as the endpoint for local control, where many patients can be salvaged by further TLM procedures, and or radiation while preserving the larynx. In comparison, repeat RT for local recurrence is generally not an option and many of these patients are salvaged by a total laryngectomy, contributing to reduced laryngeal preservation.

There were discrepancies between our analysis of oncologic outcomes of TLM versus RT in early glottic cancer and those reported by earlier systematic reviews. As previously highlighted, this may be the result of inclusion of new literature or methodological differences. Thus, if we consider just the recent systematic reviews that analyzed two-armed studies [5, 6, 32], it would appear that TLM has increasingly favourable oncologic outcomes over time. This is consistent with our findings of improved overall survival and disease-specific survival rates in TLM over RT, which is not described in earlier systematic reviews [5, 32]. Our study also had strict inclusion criteria, and thus we excluded some studies from our analysis that were included in earlier systematic reviews. For instance, three earlier reviews [5, 6, 32] included a study by Spector et al. [35], however, this study did not meet our inclusion criteria as a portion of the TLM cohort received treatment with a KTP laser. Furthermore, our study included reported event numbers for each oncologic outcome, whereas earlier systematic reviews may have extrapolated event numbers from reported Kaplan-Meier percentages. For instance, Mo et al. state that they “converted the percentages into event numbers so as to analyze the combined values of different studies.” We did not back-calculate event numbers from actuarial data in this manner to reduce potential error in our analysis. As a result, we excluded a study by Dinapoli et al. [36] and some oncologic outcome data from Rosier et al. [24], that was included in earlier reviews [5, 6]. Lastly, we excluded any studies that grouped additional stages of glottic cancer into oncologic outcomes. Thus, we excluded a study by Osborn et al. [37] that was included in earlier reviews [5, 6] as they combined Tis and T1 patients in the TLM and RT cohorts.

### Limitations

Inherently, the quality of a systematic review is limited by the quality of available literature. There were no available RCTs that met our inclusion criteria. All included studies were observational non-randomized cohort studies, the vast majority retrospective, with two prospective by design. Non-randomization may produce selection bias when allocating patients to treatment cohorts. Nine studies included in our review specified potential sources of selection bias within their methods (Table 1). Most notably, some allocated by tumor characteristics, including favouring RT for stage T1b [19, 24], increase tumor depth [22], poor tumor visualization [12, 19], or anterior commissure involvement [12, 21, 23, 24]. Allocating patients to treatment groups by key disease characteristics will ultimately create cohorts with significantly different baseline characteristics, and confound results. These studies suggest that the potential selection bias does not create significant differences in cohorts, however, just one [25] reported a statistical analysis of cohort characteristics. Three studies also treated T1 glottic cancer patients with RT years before beginning TLM therapy [16, 21, 29]. Although unintentional, this can create bias since treatment outcomes are undoubtedly impacted over time by a plethora of factors, including technological advances in health care, surgical skill, and provider experience. In contrast, the remaining seven studies did not report specific selection bias but acknowledged that patient allocation was non-randomized. Authors explained that although patients were counselled on the pros and cons of both therapies, final allocation was ultimately patient preference (Table 1). For example, Stoeckli et al. [19] report that: “advantages of laser surgery, which were explained to the patients, consisted of the single stage and short duration of the definitive treatment, the possibility of histologic examination of the resection margins, and the preservation of radiotherapy for recurrences or future second primary tumors.” Evidently, the manner in which treatment options are presented will strongly influence therapy selection and can ultimately introduce selection bias.

Variability was also present in treatment administration. For instance, there was little consensus among reported ELS types of cordectomies, poor reporting on the use of specific cancer staging systems, and just four studies [11, 14, 24, 25] reported parameters used for laser surgery such as power and spot size. There was also some variability in the RT methods, with some studies using a 4-MV or 18-MV linear accelerator [24, 27], x-rays [28], or a Co60 unit [24, 27, 28]. The discrepancies in treatment methods can introduce performance bias, impacting the interpretability of outcomes. Despite this, our meta-analysis revealed little heterogeneity between included studies when assessing oncologic outcomes for overall survival, disease-specific survival, and laryngeal preservation. Therefore, although quality of included studies must be considered, we concluded that TLM has favourable survival outcomes.

The optimal therapy in early glottic cancer will ultimately have superior survival outcomes, functional outcomes, and favourable cost utility. Here we demonstrate that TLM has favourable survival outcomes, however we do not assess functional outcomes or cost utility. A previous randomized trial of 60 men with T1 disease found that RT had favourable voice-outcomes compared to TLM, including less-hoarseness-related inconvenience at 2 years follow-up [38]. In contrast, a meta-analysis published shortly after by Du et al. [7] suggests that although they did not find significant differences in the Voice Handicap Index (VHI), jitter, or shimmer, TLM had preferable fundamental frequency values over RT. Currently, there is uncertainty as to which treatment modality has favourable functional outcomes given the paucity of research investigating this question. In terms of cost effectiveness, a recent study by Prettyjohns et al. [39] used a Markov decision model to compare cost utility and quality-adjusted life years (QALYs) of TLM and RT in early glottic cancer. They concluded that TLM was a cost-effective strategy with greater QALYs in T1a laryngeal cancers, however, there was uncertainty in T1b–T2 laryngeal cancers.

Additional research, specifically prospective RCTs will be required to address these uncertainties. Unfortunately, performing an RCT that directly compares TLM and RT may be difficult due to a number of factors, including ethical considerations and inherent institutional bias. Several attempted RCTs investigating TLM vs RT have been abandoned due to poor patient recruitment [8]. If RCTs are not possible, future studies may investigate this question using complex statistical models, such as Monte Carlo simulations, to assess probabilities of oncologic and functional outcomes with either modality.

## Conclusions

Our systematic review and meta-analysis examined studies that directly compared oncologic outcomes in T1 glottic cancer with a primary treatment of TLM or RT. There were no eligible RCTs, and all studies included in our analysis were observational cohort studies, with a level II to level III evidence rating. In addition, there was some variability in treatment methods and non-randomized patient allocation to treatment groups, creating bias. As a result, these results should be interpreted with a degree of caution.

Despite limitations, our findings demonstrated that TLM was associated with favourable outcomes in terms of overall survival, disease-specific survival and laryngeal preservation. There was no difference in associated local control between TLM and RT after the initial TLM procedure. The ability to salvage patients who recur after their initial TLM procedure with repeat TLM or radiation may explain the associated improvement in overall survival, disease specific survival and laryngeal preservation despite the associated equivalence in initial local control.

Overall, our analysis has contributed to improving our understanding of optimal management in early glottic cancer. Determining optimal therapeutic management in glottic cancer must also consider the availability of the therapy, patient preference, cost utility, and inherent advantages of each modality. Ultimately, well-designed prospective and multicentre RCTs or studies using statistical modelling will be required to provide higher quality evidence in addressing the remaining uncertainties, corroborating the efficacy of TLM in early glottic cancer, and establishing clinical guidelines.

## Data Availability

All data generated or analysed during this study are included in this published article.

## Abbreviations

AC: Anterior commissure
AJCC: American Joint Committee on Cancer
CI: Confidence interval
OR: Odds ratio
QALYs: Quality-adjusted life years
RCT: Randomized-control trial
RT: Radiotherapy
TLM: Transoral laser microsurgery
UICC: Union for International Cancer Control
